# De-differentiation of papillary thyroid carcinoma into squamous cell carcinoma in an elderly patient: A case report

**DOI:** 10.1097/MD.0000000000019892

**Published:** 2020-04-17

**Authors:** Yotsapon Thewjitcharoen, Sirinate Krittiyawong, Siriwan Butadej, Soontaree Nakasatien, Somsong Polchart, Pairoj Junyangdikul, Auchai Kanchanapituk, Thep Himathongkam

**Affiliations:** a Diabetes and Thyroid Center, Theptarin Hospital; b Division of Anatomical and Clinical Pathology, Samitivej Srinakarin Hospital, Bangkok, Thailand.

**Keywords:** de-differentiation, papillary thyroid carcinoma, squamous cell carcinoma

## Abstract

**Rationale:**

The unpredictability of thyroid cancer can be striking, as the disease may rapidly progress to death in some individuals. Herein, we reported a rare case of aggressive papillary thyroid cell carcinoma (PTC) in an elderly patient de-differentiated into squamous cell carcinoma (SCC).

**Patient concerns:**

We describe a case of a 79-year-old Thai woman presented with hoarseness and neck mass for 2 months and she had been diagnosed with a 3-cm papillary thyroid carcinoma (PTC) in the right side of the thyroid gland. Later on PTC de-differentiated into SCC within 3 years after initial presentation.

**Diagnosis:**

De-differentiation from papillary thyroid carcinoma to squamous cell carcinoma.

**Interventions:**

The patient underwent a total thyroidectomy at the initial hospital and received high dose radioactive iodine (RAI) treatment at our hospital 1 month following the surgery and then was lost to follow-up. Two years later she came back with new development of right solid-cystic neck mass which was found to be recurrent PTC. A radical neck dissection was done and another high dose RAI treatment was given. However, she developed recurrent mass with tenderness at the site above previous solid cystic mass 6 months later. Re-exploration of the neck mass revealed an inflamed midline mass 2 cm with enlarged right lateral cervical lymph nodes.

**Outcomes:**

A histopathological examination of the midline neck mass showed poorly differentiated SCC with lymphatic invasion. The intermingling of two morphologically distinct tumors, a typical PTC and a poorly differentiated SCC, had been identified in 1 out of 14 excised cervical lymph nodes. The patient underwent external beam radiation without chemotherapy. She is still in stable condition at 18 months post-treatment.

**Lessons:**

This case clearly demonstrated that SCC transformed from a pre-existing PTC. The clinician should consider a possible transformation of papillary thyroid cancer into more aggressive histological types in elderly patients who present with rapidly progressive clinical behavior. However, some patients could have long-term survival if the tumor did not transform into anaplastic thyroid cancer.

## 1 Introduction

Papillary thyroid carcinoma (PTC) is the most common thyroid cancer with its incidence having doubled over the past 2 decades from a widespread use of imaging to detect subclinical thyroid nodules rather than clinically significant PTC.^[1]^ Most patients with PTC have excellent prognosis with a 10-year survival rate of over 90%.^[2]^ However, the clinical behaviors of PTC vary and are difficult to predict in some patients especially in very elderly patients (>75 years).^[3]^ Previous studies demonstrated that initial surgery approach, tumor size, extra-thyroid invasion, lymph node metastases, and histological subtype are related to the cancer recurrence,^[4,5]^ but reliable markers to predict prognosis and seek intervention at early stage for aggressive PTC patients are not available yet in routine clinical practice.

De-differentiation from well-differentiated thyroid cancer into poorly differentiated thyroid cancer and/or anaplastic thyroid carcinoma is frequently reported with dismal prognosis (<1 year in most cases).^[6]^ However, the occurrence of squamous cell carcinoma (SCC) inside thyroid gland is uncommon condition because the normal thyroid gland has no squamous epithelial cells. Once SCC has been found from fine needle aspiration results or pathological specimens of thyroid gland, primary SCC versus secondary SCC needs to be differentiated from their diverse clinical and biological behaviors.^[7]^ PTC associated with SCC in the same lesion is an extremely rare condition in which collision tumor from other sites needs to be excluded.^[8,9]^ Herein, we reported a rare case of aggressive PTC in an elderly patient that de-differentiated into SCC in which the intermingling of 2 morphologically distinct tumors typical PTC and poorly differentiated SCC had been identified in only 1 out of 14 excised cervical lymph nodes. The patient is still in stable condition at 18 months post-diagnosis. An informed written consent was obtained from the patient for publication of this case report and accompanying images, which have been approved by the ethics committee of Theptarin Hospital, Bangkok, Thailand.

## 2 Case presentation

A 79-year-old Thai woman presented with hoarseness and neck mass. She had been diagnosed with a 3-cm PTC in right side of the thyroid gland 2 months earlier. Her medical conditions included hypertension and heart failure with preserved ejection fraction. The patient underwent a total thyroidectomy at the initial hospital and received a high dose radioactive iodine treatment (150 mCi) at our hospital 1 month following the surgery and then was lost to follow-up. Preoperative serum thyroid hormone levels were within normal range and thyroid auto-antibodies were negative. No details on initial thyroid surgery and pathological report had been obtained.

**Figure 1:**
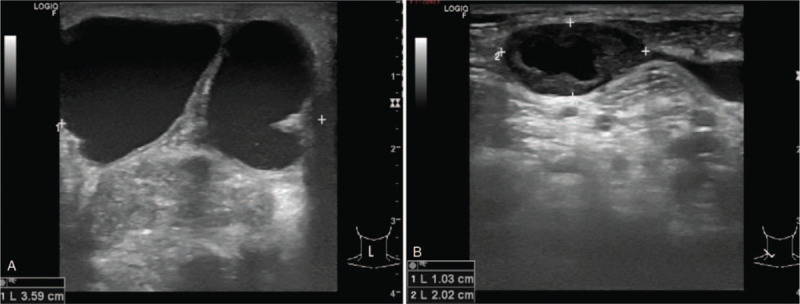
(A) Neck ultrasonography at the last presentation showed heterogeneous echogenicity of solid lesion with multiple internal calcifications at right thyroid bed (size 2.1 × 1.6 × 2.8 cm with adjacent complex multilocular cystic lesion with septations and (B) enlarged right cervical lymph node with absent fat hilum.

Two years later, she came back again with new development of a 3-cm right solid-cystic neck mass which was found to be recurrent PTC based on aspiration cytology. There was no evidence of distant metastases and there were no other obvious lymph node metastases in the neck. Radical neck dissection was done at our hospital and the second high dose radioactive iodine treatment was given (a total accumulative dose of 300 mCi). Pathological report revealed classical type of PTC without angiolymphatic invasion in excised tumor. However, she developed recurrent mass with tenderness at the site above previous solid cystic mass 6 months later. Additional work-up by neck ultrasound showed heterogeneous echogenicity of solid lesion with multiple internal calcifications at right thyroid bed (size 2.1 × 1.6 × 2.8 cm) with adjacent complex multilocular cystic lesion with septations and enlarged cervical lymph node with absent fat hilum (Fig. 1).

**Figure 2:**
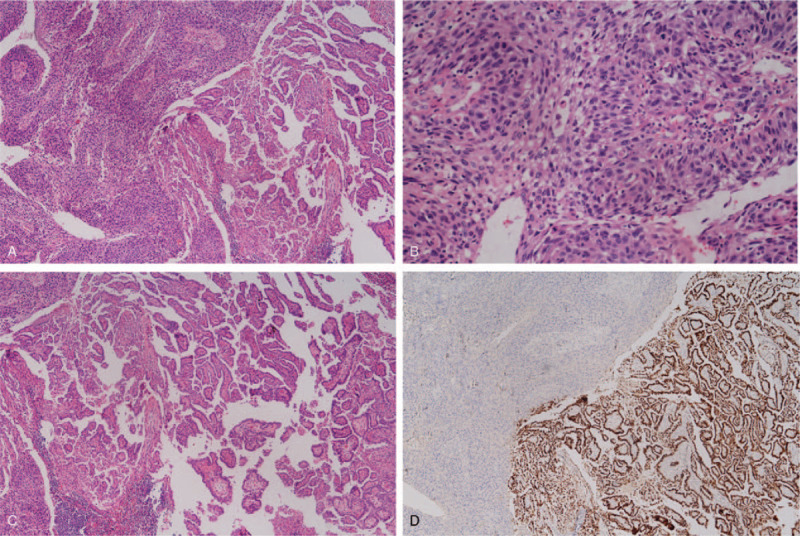
(A) Lymph node metastasis revealing 2 different histological patterns, 20×. (B) A high-power view of tumor cells with squamous cells type, moderate to poorly differentiated, 400×. (C) A group of tumor cells with classical type of papillary carcinoma. (D) Immunohistochemical staining for TTF-1 is diffusely positive in the tumor cells with papillary carcinoma morphology. TTF-1 = thyroid transcription factor 1.

**Figure 3:**
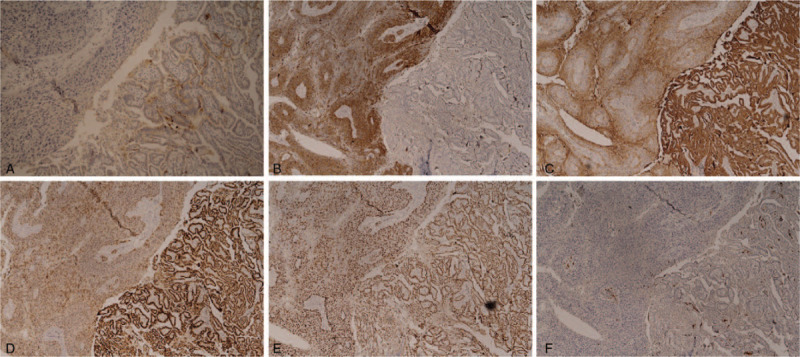
Results of various immunohistochemical staining in a dissected lymph node. (A) Thyroglobulin positive in PTC component only. (B) CK 5/6 positive in SCC component only. (C) PAX 8 positive in both PTC and SCC components. (D) CK19 positive in both PTC and SCC components. (E) P63 positive in both PTC and SCC components. (F) P16 negative in both PTC and SCC components. CK = cytokeratin, PTC = papillary thyroid carcinoma, SCC = squamous cell carcinoma.

Re-exploration of neck mass revealed inflamed midline mass 2 cm with enlarged right lateral cervical lymph nodes. A pathological examination of midline neck mass showed poorly differentiated SCC with lymphatic invasion. The intermingling of 2 morphologically distinct tumors, a classical PTC and a poorly differentiated SCC, had been identified in 1 out of 14 excised cervical lymph nodes as shown in Figure 2. Immunohistochemistry revealed P63 (a well-known marker of squamous differentiation), and paired-box gene 8 positivity in both PTC and SCC components suggesting a transformation process, not a collision tumor. While thyroglobulin was positive in PTC component only, diffuse cytokeratin 5/6 showed positive staining in only SCC component. Results of various immunohistochemical staining in a dissected lymph node are summarized in Figure 3. No molecular studies (BRAF and TERT mutation) were done.

Postoperative course was unremarkable and the patient was treated with a course of external beam radiation (40 Gray in 2 Gray per fraction) without chemotherapy because of her advanced age and co-morbidities. Concerned over airway protection, a prophylactic tracheostomy was done. The latest surveillance imaging done at 18 months after the last surgery revealed evidence of recurrent disease in the neck but the patient decided on watchful waiting without further treatments. She is still in stable condition at 18 months after the last operation.

## 3 Discussions

Patients with PTC generally have very good prognosis but risks of lifetime recurrence vary (up to 25%) depending on lymph node metastases at the time of initial surgery, size of tumor, histological subtypes, and adequacy of tumor resection.^[10]^ De-differentiation into poorly differentiated cancer or anaplastic cancer occurred in the only minority of patients which causes loss of iodine uptake ability.^[11]^ From a histopathological point of view, our patient demonstrated an unexpected finding of the development of SCC inside the lymph node of PTC metastasis. The final pathological diagnosis of PTC de-differentiated into SCC is important because treatment and prognosis differ from those of pre-existing well-differentiated thyroid cancer.

Apart from the de-differentiation process, the differential diagnosis of SCC inside the thyroid gland included primary SCC, metastatic disease from head and neck cancer, invasion from local structures, and the development of spindle cell squamous carcinoma which is a subtype of anaplastic thyroid carcinoma.^[12–14]^ Based on clinical course of our patient and an immunohistochemistry staining, our patient clearly demonstrated that SCC transformed from a pre-existing PTC. The only identified SCC in 1 from 14 excised cervical lymph nodes allowed us to reach the correct diagnosis of PTC associated with SCC. The diagnostic skills of pathologists are extremely important in treating clinicians to handle difficult thyroid cases.^[15]^ Poorly differentiated thyroid carcinomas are a subset of thyroid cancer which is an intermediate phase between well-differentiated thyroid cancer and anaplastic thyroid cancers (ATC). In the past decade, the discovery of genetic landscape of thyroid carcinogenesis and progression helped to deepen our understanding of the complex and diverse clinical and biological behaviors of differentiated thyroid cancer.^[16]^ The progression to more aggressive behavior may be predicted based on both histological subtypes and molecular signatures. Subtypes of PTC including the tall cell, columnar cell, diffuse sclerosing, and oncocytic variants tend to be more aggressive and have higher risks of recurrence and metastases. However, in contrast to the previous reports of tall cell variant of PTC de-differentiated into SCC,^[17–19]^ our case demonstrated the classical type of PTC as a previously reported case of an elderly Korean patient.^[20]^ Therefore, the unpredictable nature of PTC especially in very elderly patients should be kept in mind and lifelong follow-up is required in all cases of thyroid cancers.

A recent study revealed that PTCs harboring Telomerase (TERT) promotor mutation are more likely to transform into ATC.^[21]^ A study from Asian patients showed that coexistence of TERT and BRAF mutations was found in 13% of PTCs and was significantly associated with older age and advanced stage compared with the group negative for either mutation.^[22]^ Therefore, identification of these mutations in routine clinical practice might have a role to delineate high-risk patients and offer intervention at early stage for those aggressive PTC patients. A previous study showed that the presence of identical mutation of BRAF gene in both primary thyroid tumor and metastasis site might help to exclude tumor to tumor metastasis.^[23]^ Unfortunately, both TERT and BRAF mutational analysis did not perform in our case. Future studies should be done to clarify the molecular pathway involved in the de-differentiation process.

As the tumor cells de-differentiated, cancer becomes resistant to the traditional therapeutic strategies, and prognosis worsens significantly. Optimal management requires balance between the risk of recurrence and adverse effects from treatment. After having completed surgical resection, these patients should ideally be offered adjuvant radiotherapy or adjuvant chemoradiotherapy. However, there are no standard chemotherapy guidelines for SCC due to de-differentiation from its rarity. Due to advanced age and co-existence of co-morbidities, our patient decided on watchful waiting without further treatments after adjuvant radiation. In contrast to primary SCC or ATC, long-term survival can be expected for some patients with poorly differentiated thyroid cancer if they undergo locally curative surgery and do not have distant metastasis or an undifferentiated component in the tumors.

In conclusion, clinicians should consider a possible transformation of PTC into more aggressive histological types in elderly patients who present with rapidly progressive clinical behavior. This case provides further evidence that classical subtype of PTC can transform into SCC and it is important to consider this possibility in the presence of SCC inside the thyroid gland. The de-differentiation process from well-differentiated thyroid cancer portends worse prognosis but longer survival might be obtained if a complete surgical resection could be achieved. Further research and more case series are needed to determine a novel targeted therapy based on molecular characterization of the tumor.

## Acknowledgments

The authors wish to thank Dr. Tinapa Himathongkam for excellent language editing and the staff of Theptarin Hospital for all their support and help.

## Author contributions

**Conceptualization:** Yotsapon Thewjitcharoen.

**Investigation:** Yotsapon Thewjitcharoen, Sirinate Krittiyawong, Pairoj Junyangdikul, Auchai Kanchanapituk.

**Supervision:** Auchai Kanchanapituk, Thep Himathongkam.

**Writing – original draft:** Yotsapon Thewjitcharoen.

**Writing – review & editing:** Sirinate Krittiyawong, Siriwan Butadej, Soontaree Nakasatien, Somsong Polchart.

Yotsapon Thewjitcharoen orcid: 0000-0002-2317-4041.
